# Do cognitive activities during neuropsychological testing trigger seizures?

**DOI:** 10.1111/epi.18429

**Published:** 2025-04-28

**Authors:** Sophia Wismeth, Mostafa Badr, Christoph Helmstaedter, Juri‐Alexander Witt, Rainer Surges

**Affiliations:** ^1^ Department of Epileptology University Hospital Bonn Bonn Germany

**Keywords:** cognitive activity, memory, neuropsychological testing, onset zone, seizure occurrence

## Abstract

**Objective:**

Previous studies have suggested that stress precipitates seizures and that cognitive exertion may increase brain excitability, thereby possibly contributing to seizure occurrence during neuropsychological examinations. The present study investigates whether specific, standardized cognitive activities are linked to seizure occurrence.

**Methods:**

Two thousand neuropsychological examinations including the Verbal Learning and Memory Test (VLMT) and Diagnosticum für Cerebralschädigung–Revised (DCS‐R) were retrospectively evaluated, and occurrence of seizures during neuropsychological testing was assessed. In addition, patient characteristics and epilepsy features were collected from electronic patient charts. Statistical analysis was performed using chi‐squared test and linear mixed regression models.

**Results:**

Data of 1444 patients (age = 46 ± 17 years, 48% female) were included in the study, of whom 95% displayed focal epilepsy. Seizures occurred in 36 (1.8%) of 2000 neuropsychological examinations in 34 (2.4%) of the 1444 patients. Test‐related seizures were observed only in patients with focal epilepsy, predominantly during memory tests (58%) and most frequently during VLMT (33.3%) and DCS‐R (19.4%). A significant association was found between seizure occurrence during VLMT and a seizure onset zone in the right and left temporal lobe, whereas no such association was identified with seizures occurring during DCS‐R. No other features were linked with seizure occurrence during testing.

**Significance:**

The occurrence of seizures during neuropsychological examinations is very rare. Our data do not support the notion that specific cognitive activities favor acute onset of seizures but rather suggest a coincidental relationship.

**Key points**

- The occurrence of seizures during neuropsychological examination was very rare.
- Seizures predominantly occurred during memory‐related tasks.
- There was no increased occurrence of seizures during tests targeting a function in the seizure onset zone.
- The relationship between acute onset of seizures and testing is likely to be coincidental.

## 1 INTRODUCTION

Epilepsy is one of the most frequently diagnosed neurological disorders worldwide,^1^ affecting approximately .5%–1% of the general population over their lifetime.^2^ As a chronic neurological condition, epilepsy has a profound impact on the cognitive, psychological, and social aspects of people with epilepsy (PWE).^3^ The uncontrolled occurrence of seizures considerably contributes to the burden of patients' suffering.^4^ Despite the use of antiseizure medication (ASM), more than 30% of PWE continue to experience seizures.^5^ Therefore, elucidating the mechanisms underlying seizure induction is not only of great scientific interest, but also of critical importance for the initiation of appropriate therapy. Many PWE report that seizures can be triggered by specific factors, with stress being the most frequent.^6^ Acute stress (minutes to hours in duration) such as in examination situations or other assessment environments is specifically identified as a contributor to seizure induction.^7^ One area of interest within epilepsy research is the potential for cognitive exertion to act as a trigger for seizures. Neuropsychological examinations, which are essential for the assessment of cognitive function in PWE, often require intense cognitive effort. These assessments evaluate various cognitive domains, including memory, attention, language, and executive functions, which provide crucial information for diagnosis, treatment planning, and monitoring disease progression and treatment effects. This prompts the questions of whether there is an increased incidence of seizures linked to clinically motivated neuropsychological examinations and if so, whether the occurrence of epileptic seizures is related to epilepsy‐specific and patients' characteristics and whether cognitive activity, rather than stress alone, particularly favors the occurrence of seizures.

A few studies have already explored the relationship between cognitive activity and epilepsy. Some studies have reported increased interictal spike activation during specific cognitive tasks.^8^, ^9^ Generally, a bidirectional model is predominantly assumed.^10^ On one side, epileptic seizures can cause temporary and reversible negative effects on cognition (ictal and postictal cognitive dysfunction).^11^, ^12^, ^13^ On the other side, certain cognitive or behavioral activities may either trigger or prevent seizures.^14^, ^15^ Although the influence of epileptic activity on cognitive activity is rather undisputed, the extent to which cognitive activity affects epileptic activity remains largely unexplored. To date, a single study reported that 18 (10%) of 185 PWE undergoing presurgical assessment experienced a seizure during neuropsychological testing, suggesting that the occurrence of seizures is promoted by cognitive activity and that the tasks that typically engage the brain regions of the seizure onset zone (SOZ) increase the likelihood of epileptic seizures.^16^

Here, we retrospectively analyzed 2000 neuropsychological examinations of 1444 patients, with the aim of assessing the possibility of triggering epileptic seizures by cognitive testing and providing robust evidence on whether cognitive activity during neuropsychological testing increases the likelihood of seizures. Furthermore, we sought to determine whether the occurrence of seizures during testing is associated with epilepsy‐ and patient‐specific characteristics. Finally, understanding these dynamics is crucial for several reasons. First, it will inform the design and implementation of neuropsychological assessments, ensuring they are both effective and safe for PWE. Second, it will broaden our understanding of seizure triggers, allowing the development of personalized management plans that minimize seizure risk. Finally, our findings will provide insights into the neurobiological mechanisms underlying seizure induction, paving the way for future research in this domain.

## 2 MATERIALS AND METHODS

### 2.1 Study design and population

This study retrospectively evaluated data of PWE undergoing neuropsychological examinations between February 2018 and March 2024 in the Department of Epileptology at the University Hospital of Bonn. Inclusion criteria were all neuropsychological examinations fully completed on patients with a confirmed diagnosis of epilepsy in accordance with the International League Against Epilepsy (ILAE) criteria.^17^ Assessments that were incomplete and patients with an unconfirmed epilepsy diagnosis were excluded. The patient data were extracted from the medical records using a standardized data form. To ensure data quality and consistency, the first 200 datasets were validated through double entry after data collection was completed. Information regarding the neuropsychological tests and their results were obtained from the clinic's test database. Additionally, the test sheets and handwritten notes during testing were analyzed. Patient‐ and epilepsy‐specific characteristics were extracted from electronic medical reports and electroencephalographic (EEG) recordings. The locations of the epileptic focus and seizure onset were determined by noninvasive and/or chronic invasive EEG recordings conducted during the same or previous hospital stays. Demographic data were collected, including age, gender, and handedness. The duration of epilepsy was defined as the period between the very first seizure and the date of the neuropsychological examination. Different epilepsy types were broadly categorized according to the ILAE classification as focal, generalized, combined generalized and focal, or unclassified.^18^ Likewise, the etiology was classified in accordance with ILAE classification as structural, genetic, infectious, metabolic, immune, or unknown. The SOZ was categorized as generalized, temporal, extratemporal, or unknown. For each patient, the use and number of ASMs were recorded. “Under ASM” refers to patients who received at least one ASM, whereas “reduced/no ASM” includes both patients who were temporarily taken off at least one ASM for diagnostic purposes at the time of testing and those not on any ASM.

This retrospective audit of data collected during standard clinical care was waived by the local medical ethics committee (Ethikkommission an der Medizinischen Fakultät der Rheinischen Friedrich‐Wilhelms‐Universität Bonn, No. 352/12).

### 2.2 Neuropsychological evaluation

A standardized test battery was employed for the neuropsychological examination.^19^ The evaluation consisted of (1) the anamnesis and exploration, (2) objective testing, and (3) questionnaires. The core test battery focused on material‐specific episodic memory and executive functions and was composed of the Verbal Learning and Memory Test (VLMT)^20^ and the Diagnosticum für Cerebralschädigung–Revised (DCS‐R).^21^ Depending on the clinical question, some further tests were employed in addition (see Table 1). Test indications were clinically categorized into presurgical testing, postoperative follow‐up, differential diagnosis, etiological diagnosis, limbic encephalitis diagnosis, and others. The duration of the assessments varied between approximately 70 and 120 min. The examination was conducted by experienced and trained staff. The examiners were informed about typical seizure symptoms before every assessment, so that they were able to recognize habitual seizures during testing. As a general rule in our department, the compromised part of the neuropsychological testing is redone on another day in case of a generalized or focal to bilateral tonic–clonic seizure occurring during testing. If a focal unaware seizure occurs during the active part of the testing, the test is continued approximately 1 h after seizure cessation.^22^ If a focal unaware seizure occurs in the retention interval of the memory tests, we usually try to continue the test (i.e., the delayed recall will be performed). If the results of the retrieval are outside of normal limits, the respective memory test may be repeated at another instance.

**TABLE 1:** Neuropsychological test battery.

Core test battery	VLMT (20 min)^20^ DCS‐R (20 min)^21^ EpiTrack (15 min)^23^ ○ interference test (2 min) ○ TMT‐A/B (5 min) ○ maze test (2 min) ○ verbal phonemic fluency (3 min) ○ digits backwards (WAIS‐R) (3 min) BDI (10 min)^25^
Additional tests	Edinburgh Handedness Inventory (Oldfield; 5 min)^26^ Luria sequences (3 min)^27^ Digits span (WAIS‐R; 3 min) Corsi Block‐Tapping Test (5 min) Vocabulary/intelligence: multiple‐choice word test (MWT‐B; 5 min)^28^ FPZ (15 min)^29^

Abbreviations: BDI, Beck Depression Inventory; DCS‐R, Diagnosticum für Cerebralschädigung–Revised; FPZ, Fragebogen zur Persönlichkeit bei zerebralen Erkrankungen; MWT‐B, Mehrfachwahl‐Wortschatz‐Intelligenztest, Version B; TMT, Trail‐Making Test; VLMT, Verbal Learning and Memory Test; WAIS‐R, Wechsler Adult Intelligence Scale–Revised.

In the following, we describe the most relevant tests with highest seizure occurrence:

- VLMT: VLMT is the German adaptation of the Auditory Verbal Learning Test. During this test, patients are tasked with learning and recalling a list of 15 words over seven consecutive trials, following a delayed free recall and a recognition trial after learning a second, distracting word list.
- DCS‐R: DCS‐R requires the patient to learn abstract designs. Each design consists of five lines of equal length that must be reproduced with five wooden sticks.
- EpiTrack: EpiTrack is a tool to evaluate attention and executive functions. It is based on six subtests assessing response inhibition (interference test), visuomotor speed (Trail‐Making Test‐A [TMT‐A]), mental flexibility (TMT‐B), visual motor planning (maze test), verbal phonemic fluency, and working memory (digits backward). These subtests are versions of published tests that are typically included in traditional neuropsychological assessments.^23^ Regarding the subtests of the EpiTrack, most seizures occurred during the TMT‐A/B. In the TMT‐A, the subject has to track numbers from 1 to 25 in ascending order with a pencil. In the TMT‐B, numbers and letters have to be tracked in alternating ascending order (1‐A‐2‐B‐3‐C, etc.).
- Corsi Block‐Tapping Test: In the Corsi Block‐Tapping Test, the patient is shown a series of blocks tapped by the examiner in a specific sequence. The patient is required to replicate the sequences by tapping the blocks in the same order (forward condition) or in reverse order (backward condition), while the sequences increase by length each time.^24^
- Mehrfachwahl‐Wortschatz‐Intelligenztest, Version B (MWT‐B): In the MWT‐B, the patient is presented with a series of items, each containing one real word and several pseudowords. The patient's task is to correctly identify the real word among the pseudowords.

The results of the neuropsychological examination were classified based on the presence and localization of cognitive impairment as left temporal, right temporal, bitemporal, extratemporal, global, and no impairment. In instances where seizures occurred, the final test conducted prior to the onset of the seizure was included in this retrospective analysis.

### 2.3 Statistical analysis

IBM SPSS Statistics (v29.0.2.0 [20]) was employed for the statistical analysis. The initial step involved the distribution of patient and epilepsy‐specific characteristics within the study population using descriptive statistics and frequency analyses. To avoid distortion in patient‐related characteristics caused by multiple examinations of the same individual, only the first examination for each patient during the defined period of study was included in this analysis. Subsequently, the relationship between seizure occurrence and SOZ, as well as the association between SOZ and seizure occurrence in specific tests, particularly VLMT and DCS‐R, was examined using cross‐tabulations and the chi‐squared test. Because VLMT and DCS‐R assess verbal and visual memory functions in the dominant and nondominant hemispheres, respectively, the association with seizures of right and left temporal origin was primarily investigated. Subsequently, predictors of seizure occurrence were identified using a linear mixed model. To investigate the potential bias due to multiple examinations on individual patients, these statistical analyses were also performed on the refined dataset, which excluded all multiple examinations, as described above.

## 3 RESULTS

In 2000 neuropsychological examinations conducted on 1444 patients over a 6‐year period, seizures occurred during 36 (1.8%) assessments in 34 patients. Two patients experienced a second seizure: one during the same examination and another during a subsequent assessment conducted at a later time. To identify clinical characteristics linked to seizure onset, the frequency of seizure occurrence was compared between the group of patients who experienced seizures during testing (termed the “seizure group”) and the group of patients without seizures (termed the “nonseizure group”). After excluding all repeated examinations for the analysis of patient‐related clinical characteristics, the seizure group included 23 patients. The results, categorized into patient‐related and examination‐related characteristics, are presented in Table 2.

**TABLE 2:** Demographics and clinical characteristics (patient‐related and examination‐related).

Feature	Non‐seizure group	Seizure group
*N*	1421	23
Age (yr)		
Mean	46.28 (14–89; 17.33)	47.61 (25–85; 17.51)
Sex		
M	743 (52.3%)	14 (60.9%)
F	678 (47.7%)	9 (39.1%)
Handedness		
R	1152 (81.0%)	21 (91.3%)
L	99 (7.0%)	0 (0%)
Ambidextrous	170 (12.0%)	2 (8.7%)
Onset of epilepsy (yr)		
Mean	29.96 (0–85; 20.36)	25.00 (1–80; 19.99)
Epilepsy type		
Focal	1350 (95.0%)	21 (91.3%)
Generalized	56 (3.9%)	0 (0%)
Combined	3 (0.2%)	2 (8.7%)
Unclassified	12 (0.8%)	0 (0%)
Etiology		
Structural	492 (34.6%)	12 (52.2%)
HS	165 (11.6%)	5 (21.7%)
Tumors	105 (7.4%)	3 (13.0%)
MCD	97 (6.8%)	3 (13.0%)
VM	36 (2.5%)	0 (0%)
Glial scarring	46 (3.2%)	1 (4.3%)
unknown	39 (2.7)	0 (0%)
Genetic	45 (3.2%)	0 (0%)
Infectious	6 (0.4%)	0 (0%)
Immune	274 (19.3%)	3 (13.0%)
Unknown	604 (42.5%)	8 (34.8%)
SOZ		
Generalized	61 (4.3%)	0 (0%)
Temporal	1060 (74.6%)	18 (78.3%)
Extratemporal	161 (11.3%)	4 (17.4%)
Unknown	139 (9.8%)	1 (4.3%)
Cognitive impairment		
Left temporal	231 (16.4%)	1 (4.3%)
Right temporal	367 (25.8%)	7 (30.4%)
Bitemporal	114 (8.0%)	2 (8.7%)
Extratemporal	127 (8.9%)	4 (17.4%)
Global	323 (22.7%)	8 (34.8%)
No impairment	259 (18.2%)	1 (4.3%)

Feature	Examinations without seizures (non‐seizure group)	Examinations with seizures (seizure group)
*N*	2000	36
Duration of epilepsy (yr)		
Mean	11.82 (0–72; 13.26)	13.31 (0–48; 14.96)
Test indication		
Presurgical	450 (22.9%)	13 (36.1%)
Postoperative	146 (7.4%)	0 (0%)
Differential diagnosis	58 (3.0%)	1 (2.8%)
Etiology	388 (19.8%)	4 (11.1%)
limbic encephalitis	514 (26.2%)	12 (33.3%)
Others	408 (20.8%)	6 (16.7%)
ASM		
Under stable ASM	1294 (65.9%)	22 (61.1%)
Reduced/no ASM	670 (34.1%)	14 (38.9%)
Mean	1.68 (0–6; 0.94)	1.83 (0–6; 1.13)

*Note*: Values are presented as *n* (%) or mean (range; SD).

Abbreviations: ASM, antiseizure medication; F, female; HS, hippocampal sclerosis; L, left; M, male; MCD, malformation of cortical development; R, right; SOZ, seizure onset zone; VM, vascular malformation.

### 3.1 Demographics and clinical characteristics

#### 3.1.1 Demographics

The mean age at the time of testing was 46 ± 17 years in the nonseizure group and 47 ± 18 years in the seizure group. The gender distribution in the nonseizure group was nearly balanced, with 52.3% male and 47.7% female, whereas the seizure group consisted of 60.9% male and 38.9% female subjects. In the nonseizure group 81.0% were right‐handed, 7.0% left‐handed, and 12.0% ambidextrous. In comparison, the seizure group exhibited a higher prevalence of right‐handedness (91.3%) and a notable proportion of ambidexterity (8.7%), and no individuals identified as left‐handed.

#### 3.1.2 Test indication and ASM

In the nonseizure group, assessment of cognitive performance in limbic encephalitis (26.2%) was the most common test indication, followed by presurgical evaluation (22.9%), other indications (20.8%), etiology diagnosis (19.8%), postoperative (7.4%), and differential diagnosis (3.0%). In the seizure group, presurgical evaluation (36.1%), limbic encephalitis diagnosis (33.3%), and other indications (16.7%) were the most frequent. No seizures occurred during postoperative assessments.

In the nonseizure group, 1294 (65.9%) patients were on a stable ASM regimen at the time of the neuropsychological examination, whereas 670 (34.1%) patients were on reduced ASM or without ASM, with an average of 1.68 ± .94 ASMs per patient. In the seizure group, 22 (61.1%) were on ASM at the time of the neuropsychological examination, whereas 14 (38.9%) were on reduced ASM or without medication, with average ASMs of 1.83 ± 1.1 per patient.

#### 3.1.3 Epilepsy type

Focal epilepsy was predominant in both the nonseizure group (1350, 95.0%) and the seizure group (21, 91.3%). Additionally, the nonseizure group included 56 (3.9%) generalized, three (.2%) combined, and 12 (.8%) unclassified epilepsies. In the seizure group, there were no generalized cases, two (8.7%) combined focal and generalized cases, and no unclassified cases.

#### 3.1.4 Etiology

The etiology of epilepsy was primarily structural and immune‐mediated in both groups. In the nonseizure group, there were 492 cases (34.6%) of structural, 274 cases (19.3%) of immune‐mediated, 45 cases (3.2%) of genetic, six cases (.4%) of infectious, and 604 cases (42.5%) of unknown etiology. In the seizure group, there were 12 cases (52.2%) of structural etiology, three cases (13.0%) of immune‐mediated etiology, no genetic or infectious cases, and eight cases (34.8%) of unknown etiology. Hippocampal sclerosis (HS) was the most common structural cause of epilepsy among patients with structural epilepsies, occurring in 11.6% of the nonseizure group and 21.7% of the seizure group.

#### 3.1.5 Seizure onset zone

Regarding the SOZ, the nonseizure group displayed generalized onset in 61 cases (4.3%), temporal onset in 1060 cases (74.6%), extratemporal onset in 161 cases (11.3%), and unknown origins in 139 cases (9.8%). In the seizure group, there were no cases with generalized onset of epilepsy, 18 patients (78.3%) exhibited temporal lobe onset, four (17.4%) exhibited extratemporal onset, and one (4.3%) exhibited unknown origin.

#### 3.1.6 Cognitive impairment

The results of the neuropsychological testing showed predominantly right temporal (25.8%), global (22.7%), and left temporal (16.4%) impairments in the nonseizure group. In contrast, the seizure group demonstrated a higher prevalence of global (34.8%), right temporal (30.4%), and bitemporal (8.7%) deficits.

### 3.2 Outcome

The occurrence of seizures was documented in the context of 40 of the 2000 neuropsychological examinations that were subjected to analysis. Four of the examinations were subsequently excluded from the analysis. One patient demonstrated a documented seizure frequency of 10 seizures per hour on the day of examination, resulting in in the occurrence of multiple seizures during the course of the testing. Moreover, three patients experienced seizures outside the context of the actual cognitive testing, occurring during history taking or filling out the Beck Depression Inventory. Consequently, seizures were observed in 36 examinations of 34 patients. Two patients experienced a second seizure: one during the same examination and another during a subsequent assessment conducted at a later time. Of the 34 seizures observed during testing, 15 occurred under video‐EEG monitoring and 21 without video‐EEG monitoring. In the 21 cases without EEG monitoring, the examiners recognized the typical seizure symptoms.

Seizures occurred most frequently during VLMT with 12 cases (33.3%), followed by DCS‐R with seven cases (19.4%), the TMT‐A/B with five cases (13.9%), and the Corsi, MWT‐B, and interference tasks with two cases (5.6%) each. Additionally, one seizure was observed during the verbal fluency and maze tasks with one case (2.8%) each. In four cases (11.1%), the time of seizure occurrence was unknown, as shown in Table 3.

**TABLE 3:** Frequency of Seizures During Specific Neuropsychological Tests.

Test	Examinations with seizures, *n* = 36
VLMT	12 (33.3%)
DCS‐R	7 (19.4%)
TMT‐A/B	5 (13.9%)
Corsi	2 (5.6%)
MWT‐B	2 (5.6%)
Interference	2 (5.6%)
Verbal fluency	1 (2.8%)
Maze	1 (2.8%)
Unknown	4 (11.1%)

*Note*: Values are presented as *n* (%).

Abbreviations: DCS‐R, Diagnosticum für Cerebralschädigung–Revised; MWT‐B, Mehrfachwahl‐Wortschatz‐Intelligenztest, Version B; TMT, Trail‐Making Test; VLMT, Verbal Learning and Memory Test.

Regarding the statistical analysis of the association between seizure occurrence and SOZ, using cross‐tabulations and the chi‐squared test revealed the following (Table 4). Seizures occurred in 28 examinations of patients with a temporal SOZ, whereas no seizures occurred in 1546 tests of patients with a temporal SOZ. The chi‐squared value of χ^2^ = .218 with a *p*‐value of *p* = .640 indicates no statistically significant association. Bitemporal (*p* = .277), extratemporal (*p* = .229), and left temporal (*p* = .234) SOZs also did not show any statistical significance. Seizures occurred in 14 of 465 examinations of patients with a right temporal SOZ, representing 3% of the examinations. There was a trend toward significance regarding the association between seizure occurrence during testing and right temporal SOZ (*p* = .056).

**TABLE 4:** Seizure occurrence during testing by SOZ.

SOZ	No seizure during testing, *n*	Seizure, *n* (%)	Total, *n*	Pearson chi‐squared	*p*
Temporal	1546	28 (1.8%)	1574	.218	.640
Extratemporal	201	6 (3.0%)	207	1.447	.229
Bitemporal	275	3 (1.1%)	278	1.181	.227
Left temporal	746	11 (1.5%)	757	1.415	.234
Right temporal	465	14 (3%)	479	3.658	.056

Abbreviation: SOZ, seizure onset zone.

The association between SOZ (right temporal, left temporal) and seizure occurrence during specific tests (VLMT, DCS‐R) was analyzed using cross‐tabulations and the chi‐squared test, revealing the following. For VLMT (Table 5), seizures occurred in only one of 11 examinations of patients with a left temporal SOZ (*p* = .025). Seizures occurred in six of 11 tests of patients with a right temporal SOZ (*p* = .037). For DCS‐R (Table 6), seizures occurred in three of seven assessments of patients with a left temporal SOZ (*p* = .977). Similarly, for the patients with a right temporal SOZ, seizures occurred in two of seven evaluations (*p* = .913). The linear mixed model revealed that only the type of epilepsy (focal epilepsy) was a predictor of seizure occurrence (*p* < .001).

**TABLE 5:** SOZ by seizure occurrence during VLMT.

	No seizure during VLMT, *n*	Seizure during VLMT, *n* (%)	Total, *n*	Pearson chi‐squared	*p*
SOZ					
Left temporal	754	1 (.13%)	755	5.007	.025
Right temporal	471	6 (1.26%)	477	4.369	.037

Abbreviation: SOZ, seizure onset zone; VLMT, Verbal Learning and Memory Test.

**TABLE 6:** SOZ by seizure occurrence during DCS‐R.

	No seizure during DCS‐R, *n*	Seizure during DCS‐R, *n* (%)	Total, *n*	Pearson chi‐squared	*p*
SOZ					
Left temporal	752	3 (.40%)	755	.001	.977
Right temporal	475	2 (.42%)	477	.012	.913

Abbreviation: DCS‐R, Diagnosticum für Cerebralschädigung–Revised; SOZ, seizure onset zone.

## 4 DISCUSSION

In our study, only 1.8% of the 2000 neuropsychological examinations were found to be associated with seizures. This finding represents a notably lower occurrence as compared to an assumed rate of 10%, as reported by a previous study.^16^ In line with this study, however, the majority of patients with seizures (78.3%) exhibited a temporal SOZ.

Furthermore, in our study seizures occurred predominantly during VLMT (33.3%) and DCS‐R (19.4%). Overall, 58% of seizures occurred during memory‐related tasks (VLMT, DCS‐R, Corsi, digits backward), which differs from 100% of seizures occurring during memory‐related tasks in the aforementioned study,^16^ assuming that seizure occurrence is favored by tasks targeting temporal lobe memory functions. A more likely explanation, however, is that the substantial amount of time devoted to memory testing in the total test duration contributes to this apparent link. When only the core test battery is administered, the total test duration is 65 min, with 20 min allocated to VLMT and 20 min to DCS‐R. Hence, the duration of the memory tests accounts for 61.5% of the total testing time, which increases the likelihood of seizures occurring during these assessments.

None of the patients had a documented history of thinking‐induced seizures. Assuming that triggers are rather stereotypical in reflex epilepsies, one would expect reflex seizures to occur in a greater proportion than only two of 34 patients who experienced repeated seizures at different testing times. Meanwhile, in all other individuals, seizures did not reoccur during further neuropsychological assessments, arguing against a pronounced reflex component or thinking‐induced seizures. Furthermore, thinking‐induced seizures seem more common in generalized epilepsy than in focal epilepsy.^30^, ^31^

A comparison of etiologies between the nonseizure and seizure groups reveals a higher prevalence of structural etiologies in the seizure group (52.2%). Among structural causes, HS remains the predominant etiology in both groups. In terms of cognitive impairment, patients with seizures during testing were less likely to have no functional impairment and more likely to experience global, that is, severe cognitive impairment (34.8%), followed by right temporal impairment (30.4%). There was no apparent association between cognitive impairment, memory deficit, and seizure occurrence during related cognitive tasks. Among individuals with left temporal cognitive impairment (verbal memory deficit) 27.3% experienced seizures during VLMT, and among individuals with right temporal cognitive impairment (figural memory deficit) only 20% experienced seizures during DCS‐R.

Analyzing the relationship between seizure occurrence and SOZ revealed no significant association between seizure emergence during testing and the origin of the seizures. However, the right temporal SOZ showed a potential, although not definitively significant, influence (*p* = .056) on the occurrence of seizures during testing, as previously reported.^16^ The reason patients with temporal lobe epilepsy (TLE) of the nondominant hemisphere experienced more seizures during testing remains unclear. Some speculative evidence from biofeedback studies suggests that the right hemisphere may be less capable of controlling seizure activity compared to the left hemisphere.^32^ Additionally, it has been theorized that the right hemisphere might be more susceptible to arousal disturbances.^33^

In our study, a total of seven seizures occurred during DCS‐R, with two (28.5%) of right temporal SOZ and three of left temporal SOZ (42.9%), and 12 seizures occurred during VLMT, with one (8.3%) of left temporal and six (50%) of right temporal SOZ. The former contrasts with the previously mentioned study,^16^ where 12 of 15 (80%) seizures with right temporal onset occurred during DCS‐R, whereas the latter aligns with the finding that two of four (50%) seizures with left temporal onset occurred during VLMT. The study suggested that there is a correspondence between the eliciting function (verbal/nonverbal) and the lateralization of the epileptic focus.

However, in our study, there was no association between seizure onset during DCS‐R and either right or left temporal SOZs (*p* = .913 or .977, respectively). However, there was a significant association between seizure occurrence during VLMT and both right and left temporal SOZs (*p* = .037 and .025, respectively).

Verbal memory functions targeted by VLMT are predominantly localized in the temporal lobe of the dominant hemisphere, typically the left side. If cognitive activity in the SOZ facilitated seizure onset, a higher incidence of seizures would be expected in patients with a left temporal SOZ. However, the data indicate the opposite; more patients with a right temporal SOZ experienced seizure during VLMT.

### 4.1 Strengths and limitations of the study

Inherent to the retrospective design, it might well be that a few seizures were documented or noticed by neither the examiner nor the patient. Furthermore, it was not possible to verify whether seizures without video‐EEG monitoring correlates were epileptic seizures. Additionally, the timing and duration of the tests were not documented, making it difficult to ascertain the exact testing window, which inherently carries a certain probability of seizure occurrence, especially for the extended assessment. Moreover, the time‐of‐day dependency of seizures^31^, ^32^ and the baseline seizure frequency of patients (because of unreliable reporting by patients and incomplete documentation in our medical records) were not considered. It was also not possible to determine whether patients subjectively experienced more frequent seizures under stress. Signs of stress (e.g., increase of heart rate, decrease of heart rate variability, elevated ectodermal activity) were not routinely assessed during the neuropsychological testing, so that the influence of acute stress levels on the seizure occurrence cannot be consistently determined. Nevertheless, the aforementioned factors are likely to have mixed effects on the "true" seizure occurrence during neuropsychological testing that were caused by the testing, mitigating the risk of a one‐sided bias. There is, however, a clear selection bias in our study group in favor of patients with focal epilepsies of various origins. Therefore, generalized epilepsies were much less frequent in the study group compared to their approximately 30% prevalence in all PWE,^34^, ^35^ which may explain why patients with generalized epilepsy had no seizures during neuropsychological testing in our study sample. In terms of SOZ, TLE was the most common. A distribution of 50%–73% of cases has also been documented in studies from other epilepsy surgery centers,^36^ again leading to an overrepresentation of this patient group in our study. In terms of comparability between this study and the only previous study,^16^ it should be noted that in the prior work, structural etiologies, particularly HS, were predominant. Similarly, structural etiologies are prevalent in our study population (34.9%), with HS representing a substantial proportion (33.7%). However, our study also includes a significant proportion of inflammatory, immune‐mediated epilepsies (19.3%), which received limited clinical attention in earlier years.

The strengths of our study include the large number of considered neuropsychological examinations, the comprehensive epilepsy assessment, and the systematic approach employed, which significantly reduces the impact of potential biases and strengthens the reliability and applicability of our results to the broader population of epilepsy patients.

## 5 CONCLUSIONS

Uncontrolled seizure occurrence profoundly impacts the lives of PWE. Therefore, understanding the mechanisms underlying seizure induction is not only of great scientific interest but also fundamental for effective disease management. In this study, we investigated the occurrence of seizures during neuropsychological testing. Seizures occurred in only 1.8% of neuropsychological tests, suggesting that specific cognitive activities during neuropsychological examinations are not linked to an elevated risk of acute seizure onset. However, seizures may still occur during neuropsychological testing and potentially interfere with cognitive results. Therefore, neuropsychologist should be trained in recognition and appropriate response to seizures.

## AUTHOR CONTRIBUTIONS

**Sophia Wismeth**: Conceptualization; methodology; investigation; data curation; formal analysis; writing—original draft; writing—review & editing. **Mostafa Badr**: Conceptualization; methodology; supervision; writing—review & editing. **Christoph Helmstaedter**: Conceptualization; writing—review & editing. **Juri‐Alexander Witt**: Conceptualization; writing—review & editing. **Rainer Surges**: Conceptualization; methodology; resources; supervision; writing—review & editing.

## CONFLICT OF INTEREST STATEMENT

The authors declare no conflicts of interest related to the content of this article. R.S. has received personal fees as a speaker or for serving on advisory boards from Angelini, Bial, Desitin, Eisai, Jazz Pharmaceuticals Germany, Janssen‐Cilag, LivaNova, LivAssured, Novartis, Precisis, Rapport Therapeutics, Tabuk Pharmaceuticals, UCB Pharma, and UNEEG. He is an editorial board member of *Epilepsy and Behavior* and associate editor of *Epilepsia Open*. These activities were not related to the content of this article. We confirm that we have read the Journal's position on issues involved in ethical publication and affirm that this report is consistent with those guidelines.

## Data Availability

The data that support the findings of this study are available on request from the corresponding author. The data are not publicly available due to privacy or ethical restrictions.
